# Prevalence and correlates of depression and anxiety among Chinese international students in US colleges during the COVID-19 pandemic: A cross-sectional study

**DOI:** 10.1371/journal.pone.0267081

**Published:** 2022-04-14

**Authors:** Chenyang Lin, Yuxin Tong, Yaying Bai, Zixi Zhao, Wenxiang Quan, Zhaorui Liu, Jiuju Wang, Yanping Song, Ju Tian, Wentian Dong

**Affiliations:** 1 Neuroscience Interdepartmental Program, University of California, Los Angeles, Los Angeles, California, United States of America; 2 Department of Psychological & Brain Sciences, Boston University, Boston, Massachusetts, United States of America; 3 Department of Art History and Archaeology, Columbia University, New York, New York, United States of America; 4 Department of Psychology, Ohio State University, Columbus, Ohio, United States of America; 5 Peking University Sixth Hospital, Beijing, China; 6 Peking University Institute of Mental Health, Beijing, China; 7 NHC Key Laboratory of Mental Health (Peking University), National Clinical Research Center for Mental Disorders (Peking University Sixth Hospital), Beijing, China; Post Graduate Institute of Medical Education and Research (PGIMER), INDIA

## Abstract

**Background:**

Previous studies showed that the COVID-19 outbreak increased the levels of depression and anxiety in heterogeneous populations. However, none has explored the prevalence and correlates of depression and anxiety among Chinese international students studying in US colleges during the pandemic.

**Objective:**

This study examines the prevalence of depression and anxiety among Chinese international students enrolled in US universities during the COVID-19 pandemic and identifies the associated factors, including habits, social and psychological support, sleep quality, and remote learning.

**Methods:**

Between June and July 2020, we conducted a cross-sectional study through Wenjuanxing, a web-based survey platform. Participants were recruited with snowball sampling through 21 Chinese international student associations in US universities. The survey consisted of demographic questions, the Social Support Rating Scale (SSRS), the Insomnia Severity Index (ISI), the Patient Health Questionnaire-9 (PHQ-9), the General Anxiety Disorder-7 (GAD-7), and self-constructed questions on academic performance, financial concerns, use of social media, physical exercise, and psychological support. Cut-off scores of 10 were used for both PHQ-9 and GAD-7 to determine the binary outcomes of depression and anxiety, respectively. Bivariant analyses and multivariable logistic regression analyses were performed to identify the associated factors.

**Results:**

Among 1881 participants, we found a prevalence of depression (PHQ-9 score⩾ 10) at 24.5% and that of anxiety (GAD-7 score⩾ 10) at 20.7%. A higher risk of depression was associated with recent exposure to traumatic events, agreement to pandemic’s negative impacts on financial status, agreement and strong agreement to the negative impacts of remote learning on personal relationships, and a higher ISI score. A lower risk of depression was associated with disagreement to the negative impacts of remote learning on academic performance and future careers, strong willingness to seek professional help with emotional issues, and a higher SSRS score. In addition, a higher risk of anxiety was associated with recent exposure to traumatic events, a lot of workloads, often staying up for online classes, agreement and strong agreement to the negative impacts of remote learning on personal relationships, and a higher ISI score. A lower risk of anxiety was associated with the willingness and strong willingness to seek professional help with emotional issues, and a higher SSRS score.

**Conclusion:**

This study showed a high prevalence of depression and anxiety among Chinese international students studying in US colleges during the COVID-19 pandemic. Multiple correlates—including recent exposure to traumatic events, pandemic-related financial concerns, workload, social support, remote learning, willingness to seek professional help, and sleep quality—were identified. It is critical for future studies to further investigate this student population and for universities to provide more flexible learning options and more access to psychological services.

## Introduction

Regardless of the rapid responses from healthcare and scientific communities, COVID-19 has evolved into a global pandemic, with virus variants emerging in multiple countries. Meanwhile, non-pharmaceutical governmental interventions and personal preventative practices have been gradually integrated into social norms. With the systematic administration of the COVID-19 vaccines, several countries, including China, have been able to contain the spread at a well-controlled level and are gradually recovering from previous lockdowns into a post-COVID period.

Previous studies showed that infectious outbreaks tend to trigger mental health issues, owing to additional stressors, including health-related worries, physical constraints, financial concerns, and lack of social support [1–4]. Still, the COVID-19 pandemic has raised serious concerns over its impacts on mental health among various populations [5–9].

Mental health among college students has increasingly become a subject of public concern in the past decade. Mental disorders hold major impacts on physical wellbeing and compromise cognitive functioning, thus impairing college students’ educational and career outcomes [10–12]. Among these mental disorders, anxiety and depression are the most prevalent [13]. A study from 2009 to 2015 showed that, except for bipolar disorder, bulimia, and schizophrenia, a significantly elevated prevalence of mental disorders was observed in college students [14]. A multitude of studies has also reported alarming results of an even increased rate of mental disorders among college students during the COVID-19 pandemic [15–17]. An interview survey study found that the COVID-19 outbreak increased anxiety among college students due to health-related concerns, difficulties in concentrating, sleep disruptions, decreased social interactions, and concerns over academic performance [18]. Another study also found the disintegration of daily routines and study disruptions to be risk factors for depression and anxiety during the pandemic [19]. Other risk factors, such as the amount of exercise and participation in distant learning, were identified in Chinese adolescents during the COVID surge [7], as well as the shared concerns in academic delays and financial stress among university students [20].

As a special group of US college students, Chinese international students studying in the US cope with additional stressors compared to their peers on campus. Before the COVID-19 outbreak, these students had already been confronting various challenges, including cultural differences, language acquisition, and adjustment to a new physical and social environment [21, 22]. Studies showed that, while international students demonstrated a higher prevalence of mental health issues, this population sought less help from psychological services [22]. During the pandemic, international students from China were challenged by not only the fear of their and their loved ones being at risk of exposure but also the discrimination in both China and the US [23]. Additionally, with the closure of college campuses, many of these international students started remote learning by overcoming time differences, which led to sleep disruption and social isolation.

Despite the ongoing social and economic pressure, the number of international Chinese students studying in the US has skyrocketed in the past few years, with over 370,000 in the calendar year 2020 [24]. To our knowledge, few studies have investigated the impact of the COVID-19 pandemic specifically on this student population. Therefore, this study aims to fill this knowledge gap by studying the prevalence of anxiety and depression in this population during the COVID-19 pandemic and identifying associated factors.

## Methods

### Participants

Due to logistical reasons and requirements on quarantine, subjects were recruited with snowball sampling through 21 Chinese international student associations in US colleges on WeChat from June 12th to July 14th, 2021. Data were collected using the Wenjuanxing survey platform. The necessary sample size was estimated to be 280, based on a 24% estimated prevalence of depression among Chinese domestic college students [25]. WeChat is a well-established social media in China with over a billion active users. Wenjuanxing is a WeChat-based online survey platform widely applied in survey studies [26, 27]. During the time this study was conducted, a significant portion of the students was taking online classes either in their home country or in the US.

Eligibility criteria included: 1) aged 18 and above, 2) Chinese international students currently enrolled in a US college, 3) willing to participate after informed consent. Participants with the following conditions were excluded: 1) self-reported history of severe mental illness (e.g., schizophrenia, bipolar disorder, and substance abuse) or physical illness (e.g., cancer); 2) unable to complete the survey. This study protocol was approved by the Peking University Sixth Hospital Ethics Committee. Informed consent information was provided at the beginning of the survey, and the participants were asked to read the information and click on the “agree” button to start the survey.

### Study design

This cross-sectional study aimed to investigate the prevalence of depression and anxiety among Chinese international students in US colleges and identify associated factors. The survey had a total of 60 items. We set a minimum possible total response time at 180 seconds, allowing a minimum response time of 3 seconds for each item [28]. Three filtering questions were embedded in the survey: two bogus questions and a simple math problem (e.g., “what is 31+25”). Survey responses that 1) were incomplete, 2) had lower-than-minimum total response time, and 3) contained incorrect answer(s) to the filtering questions were excluded.

The survey questions covered the following domains:

1. Demographic questions: questions on gender, age, educational level, current location, area of study, family relationships, and financial status.
2. Self-constructed single-item questions on academic performance (workload, frequency of remote learning, levels of agreement to the impacts of remote learning on personal relationships, on academic performance, and on future careers), levels of agreement to the pandemic’s effects on financial status, frequency of social media use (both Chinese and US social media), frequency of exercise, access to psychological support (attitude towards psychological services, knowledge on common mental disorders, and sources of emotional support).
3. Social Support Rating Scale (SSRS): SSRS is a broadly applied measure of social support, with its validity and reliability previously tested in Chinese populations [29, 30]. SSRS includes a total of 10 items under 3 dimensions: objective support, subjective support, and utilization of support [31]. We used a modified version of SSRS edited by Dan Ouyang, which is more suitable for college students; Ouyang’s version adapted the wording in some items and added a new item on teacher’s support, resulting in a total of 11 items under 3 dimensions [32]. Higher scores on SSRS indicate better social support.
4. Insomnia Severity Index (ISI): ISI includes 7 items adding up to a total of 21 points. Its validity has been tested in Chinese populations [33, 34]. The degree of insomnia is assessed by the final score, which is divided into 4 score ranges corresponding with levels of insomnia severity: not clinically significant (0–7), subthreshold (8–14), moderate severity clinical (15–21), and severe clinical (22–28) insomnia [35]. Generally, a higher score on ISI indicates a higher severity of insomnia.
5. Patient Health Questionnaire (PHQ-9): PHQ-9 is a depression scale applied in research and clinical settings to examine the severity of depression symptoms [36]. Its validity has been tested within various populations, including Chinese populations [37–39]. PHQ-9 consists of 9 items with a total score of 27. A cut-off value of 10 was used to measure the binary outcomes of depression.
6. Generalized Anxiety Disorder screener (GAD-7): GAD-7 is widely used for screening anxiety symptoms [40]. Its validity has been tested within both Chinese and US populations [40–42]. GAD-7 consists of 7 items with a maximum score of 21. A cut-off value of 10 was used to measure the binary outcomes of anxiety.

### Data analysis

Statistical analyses were performed using SPSS Statistics Version 25. Prevalence of depression and anxiety were calculated from binary outcomes based on cut-off scores of 10 in both PHQ-9 and GAD-7. Bivariant analyses were carried out using either two-tailed t-tests or χ2 tests as appropriate. Variables with statistical significance in the bivariant analyses were further included into multivariable logistic regression analyses, with the binary outcomes of depression and anxiety as dependent variables. Independent variables under the category of demographic characteristics were controlled as covariates. An α value of 0.05 was used in all analyses.

## Results

Responses from 1881 participants were included in the analyses (mean age = 21.39 (SD = 2.479), female n = 905, 48.1%), outputting a general prevalence of depression (PHQ-9 score ⩾ 10) at 24.5% (n = 460) and that of anxiety (GAD-7⩾ 10) at 20.7% (n = 390). The mean total scores for PHQ-9 and GAD-7 were 6.17 (SD = 6.28) and 5.10 (SD = 5.48), respectively.

Table 1 summarizes the demographic information and distribution of responses within the sample cohort. Bivariant analyses were conducted on each independent variable to the binary outcomes of depression and anxiety, using either two-tailed χ2 tests or t-tests as applicable.

**Table 1:** Demographic characteristics of the sample cohort. Bivariant analyses were performed using two-tailed t-tests or χ2 tests as appropriate.

Variables		Total (N = 1881)		Depression (PHQ-9 > = 10) (n = 460)				Anxiety (GAD-7> = 10) (n = 390)			
		N	%	N	%	X2	*P*	N	%	X2	*P*
**Gender**	**Male**	976	51.9%	253	25.9%	2.363	0.124	208	21.3%	0.412	0.521
	**Female**	905	48.1%	207	22.9%			182	20.1%		
**Education**	**Bachelor**	1302	69.2%	319	24.5%	0.461	0.794	275	21.1%	0.818	0.664
	**Master**	508	27.0%	126	24.8%			103	20.3%		
	**Ph.D.**	71	3.8%	15	21.1%			12	16.9%		
**Area of study**	**STEM**	710	37.7%	200	28.2%	9.021	**0.011**	170	23.9%	7.442	**0.024**
	**Non STEM**	839	44.6%	191	22.8%			161	19.2%		
	**Arts**	332	17.7%	69	20.8%			59	17.8%		
**Current location**	**China**	1839	97.8%	442	24.0%	7.874	**0.005**	377	20.5%	2.730	0.099
	**United States**	42	2.2%	18	42.9%			13	31.0%		
**Good family relationships**	**Strongly Disagree**	45	2.4%	16	35.6%	116.139	**<0.001**	15	33.3%	128.985	**<0.001**
	**Disagree**	40	2.1%	27	67.5%			28	70.0%		
	**Neither**	115	6.1%	62	53.9%			52	45.2%		
	**Agree**	665	35.4%	164	24.7%			145	21.8%		
	**Strongly Agree**	1017	54.1%	191	18.8%			150	14.7%		
**Source of tuition**	**Parents and Family Members**	1602	85.2%	361	22.5%	89.500	**<0.001**	304	19.0%	78.553	**<0.001**
	**Friends**	46	2.4%	36	78.3%			30	65.2%		
	**Bank Loans**	71	3.8%	29	40.8%			29	40.8%		
	**Income**	156	8.3%	31	19.9%			25	16.0%		
	**Others**	6	0.3%	3	50.0%			2	33.3%		
** **		**Mean**	**SD**	**Mean**	**SD**	**t**	***P***	**Mean**	**SD**	**t**	***P***
**Age**		21.39	2.479	21	2.291	3.85	**<0.001**	20.98	2.223	3.617	**<0.001**

Bolded values: *P* < 0.05 in the bivariant analyses.

Statistical significance was found between the risk of depression and demographic characteristics including the area of study (*P* = 0.011), current location (*P* = 0.005), family relationships (*P* < 0.001), source of tuition (*P* < 0.001), and age (*P* < 0.001). Following factors were also significantly associated with the risk of depression: recent COVID-related traumatic event(s) (*P* < 0.001), pandemic’s negative impacts on financial status (*P* < 0.001), frequency of US social media use (*P* = 0.004), frequency of exercise in the past two weeks (*P* < 0.001), the workload in the past two weeks (*P* < 0.001), frequency of staying up for online classes in the past two weeks (*P* < 0.001), impacts of remote learning on personal relationships (*P* < 0.001), and impacts of remote learning on academic performance and future careers (*P* < 0.001), willingness to seek professional help (*P* < 0.001), the amount of knowledge about common mental disorders (*P* < 0.001), number of sources for emotional support (*P* < 0.001), the Social Support Rating Scale score (*P* < 0.001), and the Insomnia Severity Index score (*P* < 0.001).

Similarly, significant associations were found between the risk of anxiety and demographic characteristics including the area of study (*P* = 0.024), family relationships (*P* < 0.001), source of tuition (*P* < 0.001), and age (*P* < 0.001). Following factors were also significantly associated with the risk of anxiety: recent COVID-related traumatic event(s) (*P* < 0.001), pandemic’s negative impacts on financial status (*P* < 0.001), frequency of US social media use (*P* < 0.001), frequency of exercise in the past two weeks (*P* < 0.001), the workload in the past two weeks (*P* < 0.001), frequency of staying up for online classes in the past two weeks (*P* < 0.001), negative impacts of remote learning on personal relationships (*P* < 0.001), negative impacts of remote learning on academic performance and future careers (*P* < 0.001), willingness to seek professional help (*P* < 0.001), the amount of knowledge about common mental disorders (*P* < 0.001), number of sources for emotional support (*P* < 0.001), the Social Support Rating Scale score (*P* < 0.001), and the Insomnia Every Index score (*P* < 0.001).

Variables with statistical significance in the bivariant analyses were included in the multivariable logistic analyses as predictor variables for the binary outcome measures of depression and anxiety (Table 2). Statistically significant variables that were categorized as demographic characteristics were controlled as covariates. The multivariable logistic regression analysis for depression demonstrated associations between a higher risk and recent exposure to traumatic event(s) (OR  = 1.653, 95% CI: 1.185–2.305), agreement to pandemic’s negative impacts on financial status (OR  =  2.000, 95% CI: 1.000–4.001), agreement (OR  =  2.052, 95% CI: 1.155–3.644) and strong agreement (OR  =  3.193, 95% CI: 1.331–7.662) to the perceived negative impacts of remote learning on personal relationships, and a higher ISI score (OR  =  1.267, 95% CI: 1.214–1.322). A lower risk of depression was associated with disagreement to the negative impacts of remote learning on academic performance and future careers (OR  =  0.549, 95% CI: 0.326–0. 925), strong willingness to seek professional help with emotional issues (OR  =  0.358, 95% CI: 0.157–0.818), and a higher SSRS score (OR  =  0.950, 95% CI: 0.929–0.970).

**Table 2:** Independent correlates of depression and anxiety by multivariable logistic regression analysis. The analysis for depression was adjusted for the area of study, current location, family relationships, source of tuition, and age; the analysis for anxiety was adjusted for the area of study, family relationships, source of tuition, and age. The frequency of US social media use, frequency of exercise in the past two weeks, and amount of knowledge about common mental disorders were not significantly associated with either the risk of depression or the risk of anxiety and were removed from the table for abbreviation purposes.

Variables		Depression (PHQ-9 score > = 10) (N = 460)				Anxiety (GAD-7 score > = 10) (N = 390)			
		Sig.	OR	95% C.I. for AOR		Sig.	OR	95% C.I. for AOR	
				Lower	Upper			Lower	Upper
**Recent exposure to traumatic event**		**0.003**	1.653	1.185	2.305	**0.003**	1.670	1.190	2.344
**Pandemic’s negative impacts on financial status**	**Strongly Disagree**	ref				ref			
	**Disagree**	0.979	1.010	0.471	2.170	0.438	1.364	0.623	2.987
	**Neither**	0.366	1.373	0.691	2.727	0.957	0.980	0.475	2.022
	**Agree**	**0.050**	2.000	1.000	4.001	0.410	1.358	0.656	2.810
	**Strongly Agree**	0.117	1.847	0.858	3.977	0.147	1.796	0.813	3.967
**Workloads in the past two weeks**	**None**	ref				ref			
	**A little**	0.152	1.522	0.856	2.704	0.087	1.696	0.926	3.106
	**Medium**	0.205	1.397	0.833	2.344	0.153	1.494	0.862	2.591
	**A lot**	0.173	1.467	0.845	2.548	**0.025**	1.927	1.084	3.425
	**Too much**	0.316	1.617	0.633	4.133	0.068	2.430	0.937	6.304
**Frequency of staying up due to remote learning (past 2 weeks)**	**Never**	ref				ref			
	**Seldom**	0.861	1.049	0.616	1.784	0.260	1.380	0.788	2.417
	**Sometimes**	0.806	0.936	0.549	1.594	0.175	1.467	0.843	2.551
	**Often**	0.228	1.406	0.808	2.449	**0.006**	2.234	1.263	3.951
	**Always**	0.716	1.162	0.519	2.602	0.198	1.699	0.757	3.812
**Negative impacts of remote learning on personal relationships**	**Strongly Disagree**	ref				ref			
	**Disagree**	0.785	0.930	0.553	1.565	0.761	0.912	0.504	1.650
	**Neither**	0.189	1.446	0.834	2.509	0.109	1.644	0.895	3.019
	**Agree**	**0.014**	2.052	1.155	3.644	**0.002**	2.645	1.414	4.949
	**Strongly Agree**	**0.009**	3.193	1.331	7.662	**0.010**	3.199	1.317	7.774
**Negative impacts of remote learning on academic performance and future careers**	**Strongly Disagree**	ref				ref			
	**Disagree**	**0.024**	0.549	0.326	0.925	0.368	0.760	0.418	1.382
	**Neither**	0.064	0.591	0.338	1.031	0.943	0.977	0.523	1.825
	**Agree**	0.618	0.867	0.495	1.519	0.304	1.387	0.743	2.590
	**Strongly Agree**	0.708	0.863	0.398	1.868	0.349	1.467	0.658	3.271
**I will seek professional help when I think I have emotional issues**	**Strongly Disagree**	ref				ref			
	**Disagree**	0.531	0.777	0.353	1.711	0.320	0.665	0.298	1.484
	**Neither**	0.093	0.523	0.246	1.114	0.052	0.467	0.217	1.007
	**Agree**	0.136	0.566	0.268	1.197	**0.050**	0.466	0.218	0.999
	**Strongly Agree**	**0.015**	0.358	0.157	0.818	**0.018**	0.360	0.154	0.838
**Social support rating scale score**		**<0.001**	0.950	0.929	0.970	**0.001**	0.964	0.943	0.986
**Insomnia severity index score**		**<0.001**	1.267	1.214	1.322	**<0.001**	1.200	1.150	1.252

Bolded values indivcates statistical significance (*P* < 0.05). *CI* indicates confidence interval. *OR* indicates odds ratio. *AOR* indicates adjusted odds ratio.

In addition, a higher risk of anxiety was associated with recent exposure to traumatic event(s) (OR  = 1.670, 95% CI: 1.190–2.344), a lot of workloads (OR  =  1.927, 95% CI:1.084–3.425), often staying up due to remote classes (OR  =  2.234, 95% CI: 1.263–3.951), agreement (OR  =  2.645, 95% CI: 1.414–4.949) and strong agreement (OR  =  3.199, 95% CI: 1.317–7.774) to the negative impacts of remote learning on personal relationships, and a higher ISI score (OR  =  1.200, 95% CI: 1.150–1.252). A lower risk of anxiety was associated with the willingness (OR  =  0.466, 95% CI: 0.218–0.999) and strong willingness (OR  =  0.360, 95% CI: 0.154–0.838) to seek professional help with emotional issues, and a higher SSRS score (OR  =  0.964, 95% CI: 0.943–0.986).

## Discussion

To our knowledge, this is the first study to investigate the prevalence of depression and anxiety and the associated factors among Chinese international students studying in US colleges during the COVID-19 pandemic. Previous studies largely focused on domestic student populations and, thus, could not account for the additional stressors experienced by international students.

Our results revealed prevalence levels of depression (PHQ-9 score ≥ 10) and anxiety (GAD-7 score ≥ 10) at 24.5% and 20.7%, respectively. These levels were similar to previous studies on Chinese college students during the COVID-19 pandemic and were lower than those found in the general population of US college students (48.14% depression, 38.48% anxiety) during the pandemic using the same measures (PHQ-9 and GAD-7) [43, 44]. Global synthesizing studies comparing Chinese college students with non-Chinese college students also found generally lower prevalence of depression and anxiety among Chinese students [15, 16]. Further investigation is needed to understand why Chinese international students experience lower risks, considering that this population may have to face additional challenges during the pandemic, including the time differences in remote learning and the discrimination in both China and the US. However, as the majority of the international students went back to China during the pandemic, where viral transmissions were more strictly controlled, they may not have to be worried about exposure to viruses as much.

We found that students in non-STEM majors generally have lower risks of depression and anxiety than those in STEM majors. Additionally, significant differences in age were also found between participants with depression or anxiety and those without, consistent with previous findings on the association between age and risks of mental disorders [44]. While many studies revealed gender differences in depression and anxiety during COVID-19 [45, 46], our results, interestingly enough, demonstrated no significant gender differences in either depression or anxiety prevalence. It is still unclear why gender gaps in depression and anxiety are missing in this student population. While a higher genetic susceptibility to depression and anxiety was shown in female [47, 48], life circumstances and cultural stressors could also explain the higher risks [49]. It is worth pointing out that, during the pandemic, social and cultural stressors, such as financial concerns and work overload, were relatively equally distributed among all international students and, thus, may reduce the gender discrepancies resulted from differences in socio-cultural circumstances. Finally, compared to students located in China, those located in the US had a significantly higher risk of depression. Many factors could contribute to such a difference, including the unfamiliarity with the new environment and more concerns over viral transmission among students located in the US.

We have also identified associated risk and protective factors in the Chinese international students studied, which should be considered in future interventions and treatments targeting this student population. Recent exposure to traumatic event(s) has been found to be associated with higher risks of depression and anxiety, echoing previous findings on the associations between depressive and post-traumatic stress symptoms in the Chinese population [44]. With the first COVID-19 outbreak observed in China, Chinese international students had to face challenges directly or indirectly related to the pandemic, such as witnessing a life-threatening experience, disruptions to family or school gatherings, or bankruptcy. Exposure to traumatic events like these is related to a wide spectrum of adverse psychological and psychiatric outcomes [49, 50]. Furthermore, consistent with the previous research on the association between pandemic’s economic impacts and mental health among various populations [51], our results showed that pandemic-related financial concerns were correlated with a higher risk of depression. This could potentially explain the finding that participants who had friends or bank loans as financial sources were at higher risks of depression and anxiety, as adverse impacts on personal finance may elevate the likelihood of students getting financial support from sources other than families.

Another significant factor to consider is remote learning. While workload still influenced stress levels, staying up for online classes due to time zone differences was significantly associated with a higher risk of anxiety among the participants. The association between sleep quality and anxiety has been well supported by the literature [52, 53]. In our study, associations were found between a higher self-reported score on the Insomnia Severity Index and higher risks of depression and anxiety, which was coherent with previous studies that showed the interactions between poor sleep quality and mental disorders [53–55]. Many participants also reported that remote learning had impaired their personal relationships, and according to our findings, such an impact, as perceived by the participants, was associated with higher risks of depression and anxiety.

We also found that higher scores on the social support rating scale (SSRS) were associated with lower risks of depression and anxiety, indicating that sufficient social support acts as a protective factor among this population. This is consistent with previous research that showed the positive association between level of social support and mental health status [31, 56]. Additionally, willingness to seek professional support was associated with lower risks of depression and anxiety. Therefore, it is critical for universities to develop more comprehensive community networks for students to feel more supported both socially and academically. School psychological services should also provide more instructions on mental health maintenance through workshops and social media and encourage students to seek professional help when needed by making the services more accessible.

Several limitations should be pointed out for future investigations to increase data reliability and generalizability. Firstly, as the data were collected via an online survey that was distributed through snowball sampling, a nonprobability sampling strategy, systematic biases may exist. Additionally, the study was cross-sectional and thus lacked a longitudinal comparison to explore the temporal dynamics of the prevalence and risk factors within the same population or to rule out the possibility of a transiently inflated estimate during the time of data collection. Furthermore, the survey didn’t include a variable to accurately capture the participants’ lengths of stay in the US, which could potentially be an important correlate of mental health problems. Lastly, the sample was not evenly distributed regarding the levels of education (Undergraduate, Master, Ph.D.) and current locations, with undergraduate students and students located in China dominating. Future studies may narrow down the participant inclusion criteria on these two factors to a specific subgroup or collect comparable amounts of data from the subgroups to measure the prevalence and identify risk factors among college students more accurately.

## Conclusions

We found prevalence levels of depression (PHQ-9 score ≥ 10) and anxiety (GAD-7 score ≥ 10) at 24.5% and 20.7% among 1881 Chinese international students studying in US colleges during the COVID-19 pandemic. Recent exposure to traumatic event(s), negative impacts of remote learning on personal relationships, and poor sleep quality were found to be associated with higher risks of depression and anxiety, while willingness to seek psychological services and better social support were associated with lower risks. A higher risk of depression was also associated with the pandemic’s negative impacts on financial status and the negative impacts of remote learning on academic performance and future careers. A higher risk of anxiety was also associated with workload and the frequency of staying up for online classes. It is crucial for universities to open in-person instructions, provide more flexible learning options for students with time-zone differences and provide students with more access to psychological services and information on mental health.

## Supporting information

S1 Table Variables other than demographic characteristics for the sample cohort. Bivariant analyses were performed using two-tailed t-tests or χ2 tests as appropriate. Bolded values: *P* < 0.05 in the bivariant analyses.
(DOCX)

S1 Dataset (XLSX)
