# Supplementary material for: Prevalence and correlates of depression and anxiety among Chinese international students in US colleges during the COVID-19 pandemic: A cross-sectional study
Source: PLoS One. 2022 Apr 14;17(4):e0267081. doi: 10.1371/journal.pone.0267081 (PMC9009639; doi:10.1371/journal.pone.0267081)
Supplement: S1 Table — Bivariant analyses were performed using two-tailed t-tests or χ2 tests as appropriate. Bolded values: P < 0.05 in the bivariant analyses. (DOCX) [file pone.0267081.s001.docx]

**Supporting information**

**S1 Table. Variables other than demographic characteristics for the sample cohort.** Bivariant analyses were performed using two-tailed t-tests or χ2 tests as appropriate. Bolded values: *P* < 0.05 in the bivariant analyses.

| **Variable** | | **Total (N=1881)** | | **Depression (PHQ-9 >=10) (N =460)** | | | | **Anxiety (GAD-7 >= 10) (N = 390)** | | | |
| --- | --- | --- | --- | --- | --- | --- | --- | --- | --- | --- | --- |
|  |  | **N** | **%** | **N** | **%** | **X2** | ***P*** | **N** | **%** | **X2** | ***P*** |
| **Recent exposure to traumatic event** | **Yes** | 322 | 17.1% | 136 | 42.2% | 66.487 | **<0.001** | 121 | 37.6% | 67.069 | **<0.001** |
|  | **No** | 1559 | 82.9% | 324 | 20.8% |  |  | 269 | 17.3% |  |  |
| **Pandemic's negative impacts on financial status** | **Strongly Disagree** | 128 | 6.8% | 18 | 14.1% | 46.469 | **<0.001** | 15 | 11.7% | 41.141 | **<0.001** |
|  | **Disagree** | 276 | 14.7% | 45 | 16.3% |  |  | 48 | 17.4% |  |  |
|  | **Neither** | 679 | 36.1% | 143 | 21.1% |  |  | 111 | 16.3% |  |  |
|  | **Agree** | 611 | 32.5% | 189 | 30.9% |  |  | 154 | 25.2% |  |  |
|  | **Strongly Agree** | 187 | 9.9% | 65 | 34.8% |  |  | 62 | 33.2% |  |  |
| **Frequency of Chinese social media use** | **Never** | 9 | 0.5% | 2 | 22.2% | 6.658 | 0.155 | 1 | 11.1% | 5.872 | 0.209 |
|  | **Seldom** | 91 | 4.8% | 24 | 26.4% |  |  | 26 | 28.6% |  |  |
|  | **Sometimes** | 345 | 18.3% | 101 | 29.3% |  |  | 79 | 22.9% |  |  |
|  | **Often** | 941 | 50.0% | 211 | 22.4% |  |  | 183 | 19.4% |  |  |
|  | **Always** | 495 | 26.3% | 122 | 24.7% |  |  | 101 | 20.4% |  |  |
| **Frequency of US social media use** | **Never** | 57 | 3.0% | 19 | 33.3% | 15.635 | **0.004** | 16 | 28.1% | 19.638 | **<0.001** |
|  | **Seldom** | 235 | 12.5% | 75 | 31.9% |  |  | 68 | 28.9% |  |  |
|  | **Sometimes** | 564 | 30.0% | 144 | 25.5% |  |  | 127 | 22.5% |  |  |
|  | **Often** | 798 | 42.4% | 166 | 20.8% |  |  | 136 | 17.0% |  |  |
|  | **Always** | 227 | 12.1% | 56 | 24.7% |  |  | 43 | 18.9% |  |  |
| **Frequency of exercise in the past two weeks** | **Never** | 23 | 1.2% | 10 | 43.5% | 67.798 | **<0.001** | 10 | 43.5% | 63.876 | **<0.001** |
|  | **Seldom** | 206 | 11.0% | 82 | 39.8% |  |  | 68 | 33.0% |  |  |
|  | **Sometimes** | 520 | 27.6% | 162 | 31.2% |  |  | 143 | 27.5% |  |  |
|  | **Often** | 926 | 49.2% | 165 | 17.8% |  |  | 137 | 14.8% |  |  |
|  | **Always** | 206 | 11.0% | 41 | 19.9% |  |  | 32 | 15.5% |  |  |
| **Workload in the past two weeks** | **None** | 171 | 9.1% | 56 | 32.8% | 23.805 | **<0.001** | 42 | 24.6% | 27.786 | **<0.001** |
|  | **A little** | 239 | 12.7% | 63 | 26.4% |  |  | 53 | 22.2% |  |  |
|  | **Medium** | 951 | 50.6% | 193 | 20.3% |  |  | 156 | 16.4% |  |  |
|  | **A lot** | 472 | 25.1% | 129 | 27.3% |  |  | 121 | 25.6% |  |  |
|  | **Too much** | 48 | 2.6% | 19 | 39.6% |  |  | 18 | 37.5% |  |  |
| **Frequency of staying up due to remote learning (past 2 weeks)** | **Never** | 216 | 11.5% | 61 | 28.2% | 56.248 | **<0.001** | 41 | 19.0% | 61.179 | **<0.001** |
|  | **Seldom** | 495 | 26.3% | 94 | 19.0% |  |  | 75 | 15.2% |  |  |
|  | **Sometimes** | 740 | 39.3% | 147 | 19.9% |  |  | 129 | 17.4% |  |  |
|  | **Often** | 361 | 19.2% | 127 | 35.2% |  |  | 117 | 32.4% |  |  |
|  | **Always** | 69 | 3.7% | 31 | 44.9% |  |  | 28 | 40.6% |  |  |
| **Negative impacts of remote learning on personal relationships** | **Strongly Disagree** | 329 | 17.5% | 36 | 10.9% | 216.74 | **<0.001** | 23 | 7.0% | 244.46 | **<0.001** |
|  | **Disagree** | 726 | 38.6% | 100 | 13.8% |  |  | 73 | 10.1% |  |  |
|  | **Neither** | 444 | 23.6% | 140 | 31.5% |  |  | 123 | 27.7% |  |  |
|  | **Agree** | 313 | 16.6% | 140 | 44.7% |  |  | 131 | 41.9% |  |  |
|  | **Strongly Agree** | 69 | 3.7% | 44 | 63.8% |  |  | 40 | 58.0% |  |  |
| **Negative impacts of remote learning on academic performance and future careers** | **Strongly Disagree** | 268 | 14.2% | 38 | 14.2% | 160.01 | **<0.001** | 24 | 9.0% | 176.13 | **<0.001** |
|  | **Disagree** | 722 | 38.4% | 108 | 15.0% |  |  | 83 | 11.5% |  |  |
|  | **Neither** | 448 | 23.8% | 117 | 26.1% |  |  | 105 | 23.4% |  |  |
|  | **Agree** | 351 | 18.7% | 143 | 40.7% |  |  | 129 | 36.8% |  |  |
|  | **Strongly Agree** | 92 | 4.9% | 54 | 58.7% |  |  | 49 | 53.3% |  |  |
| **I will seek help from professionals when I think I have emotional issues** | **Strongly Disagree** | 67 | 3.6% | 29 | 43.3% | 68.718 | **<0.001** | 25 | 37.3% | 57.665 | **<0.001** |
|  | **Disagree** | 168 | 8.9% | 68 | 40.5% |  |  | 58 | 34.5% |  |  |
|  | **Neither** | 410 | 21.8% | 127 | 31.0% |  |  | 109 | 26.6% |  |  |
|  | **Agree** | 883 | 46.9% | 182 | 20.6% |  |  | 150 | 17.0% |  |  |
|  | **Strongly Agree** | 353 | 18.8% | 54 | 15.3% |  |  | 48 | 13.6% |  |  |
| **I have a fair amount of knowledge of common mental disorders** | **Strongly Disagree** | 54 | 2.9% | 18 | 33.3% | 30.809 | **<0.001** | 14 | 25.9% | 19.671 | **<0.001** |
|  | **Disagree** | 170 | 9.0% | 66 | 38.8% |  |  | 51 | 30.0% |  |  |
|  | **Neither** | 568 | 30.2% | 149 | 26.2% |  |  | 134 | 23.6% |  |  |
|  | **Agree** | 828 | 44.0% | 167 | 20.2% |  |  | 142 | 17.1% |  |  |
|  | **Strongly Agree** | 261 | 13.9% | 60 | 23.0% |  |  | 49 | 18.8% |  |  |
|  |  | **Mean** | **SD** | **Mean** | **SD** | **t** | **P** | **Mean** | **SD** | **t** | **P** |
| **Number of sources for emotional support** | | 3.27 | 1.34 | 2.77 | 1.23 | 9.401 | **<0.001** | 2.78 | 1.27 | 8.19 | **<0.001** |
| **Social support rating scale score** | | 39.9 | 8.24 | 35.3 | 7.61 | 14.55 | **<0.001** | 35.3 | 7.81 | 12.79 | **<0.001** |
| **Insomnia severity index score** | | 4.52 | 3.56 | 8.7 | 4.168 | -20.66 | **<0.001** | 7.17 | 3.88 | -17.94 | **<0.001** |
